# Study on Dynamic Monitoring of Wire Rope Tension Based on the Particle Damping Sensor

**DOI:** 10.3390/s19020388

**Published:** 2019-01-18

**Authors:** Gaoyang Lei, Guiyun Xu, Xiaoguang Zhang, Yayun Zhang, Zhenyue Song, Wentao Xu

**Affiliations:** School of Mechatronic Engineering, China University of Mining & Technology, Xuzhou 221116, China; lgy767081515@163.com (G.L.); doctorzxg@163.com (X.Z.); 18052127955@163.com (Y.Z.); cumtsongzhenyue@163.com (Z.S.); TS17050077A3@cumt.edu.cn (W.X.)

**Keywords:** mine hoist, wire rope tension, particle damping sensor, damping vibration and energy dissipation

## Abstract

Real-time monitoring of wire rope tension is of great significance to the safe operation of mine hoist. Due to the longitudinal and lateral coupling vibration of wire ropes during the operation of hoist, there are high frequency components in measured tension signals of wire ropes, which cannot effectively characterize the actual lifting load. To overcome this problem, a particle damping sensor with a vibration dissipation function is designed in this paper. Multilayered steel balls are placed into the cylindrical cavity of the sensor. Damping vibration and energy dissipation will occur when the sensor is subjected to external excitation. Then, to obtain the optimal sensor characteristics, relevant parameters of the particles and the spoke structure are simulated. Finally, the sensor based on the optimized parameters is manufactured and tested in a coal mine. Compared with the general pressure sensor, the particle damping sensor can effectively eliminate the influence of wire ropes vibration on tension measurement and achieve accurate measurement results.

## 1. Introduction

Multi-rope friction hoist plays a key role in the safe production and transportation of coal mine. The hoist periodically goes through acceleration, uniform speed, deceleration, crawling, and stopping in the running process. It is prone to lead to the fluctuation of wire ropes due to the non-smooth cage guide, the strong wind, and the varied weight of the tail ropes during the lifting or descending process. This may result in tension imbalance, and even serious accidents, such as wire ropes wearing, fracturing, and cage falling [1,2,3]. Therefore, the real-time and accurate tension monitoring of wire ropes is of great significance for mine safety.

Many scholars have conducted lots of work on the design of the wire rope tension monitoring system and some relevant sensors used in force measurement under different kinds of conditions. ABB Sweden invented a pin-shaft type elastic element, HAMB dynamic tension measuring device which is in series with the wire ropes, to monitor the tension of wire ropes [4]. Beus et al. [5] used a new type of force transducer to determine whether the wire ropes are slight or tight. Wang et al. [6] put forward a system scheme of dynamic wire rope tension monitoring for multi-rope hoist, and designed a sleeve-hydraulic sensor and a transmission mode of induction signals of wire ropes. Zhang et al. [7] designed a new type of acoustic filtering sensor, with a cylindrical cavity and a narrow gap which can greatly absorb vibration energy to realize accurate wire rope tension measurement. Deng et al. [8] proposed a new stress measuring sensor including a static magnetization unit made of permanent magnets and a magnetic field measurement unit to evaluate the axial stress in steel wires. Xu et al. [9] designed a wireless sensor network technology adapted to underground channel conditions, which has important theoretical and practical value for safety monitoring in underground coal mines. Chen et al. [10] designed and fabricated a miniaturized Co-based amorphous wire GMI (Giant magneto-impedance) magnetic sensor with a high sensitivity. The three-dimensional micro pick-up coil was designed and simulated with HFSS (High Frequency Structure Simulator) software to determine the key parameters. To calculate the pressure of the sensor according to the linear relationship between the center wavelength offsets and the tension of steel wire rope, Wang et al. [11] designed a fiber grating sensor to detect center wavelength offsets of fiber grating. At present, it is the most economical and practical approach to install sensors on the hydraulic balancing device of hoist to measure wire ropes tension. Normally, the approach includes the oil pressure measuring method and tension conversion pressure method [1,2]. Because of the influence of friction and oil pressure loss, it is normally difficult to achieve an accurate measurement for oil pressure with the former method. The latter one is employed to install a pressure sensor between the piston rod and the sliding block. However, the tension signals of wire ropes measured by a general pressure sensor usually contain violent fluctuation and noise, which cannot effectively reflect the actual lifting load and tension. Therefore, it is necessary to design a new type of sensor with the function of absorbing vibration and dissipating the impact load of wire ropes. 

Nowadays, particle damping is a common vibration absorption technology. It absorbs vibration and dissipates energy by inelastic collision and friction between particles and the damper wall, which has a good effect on vibration attenuation and isolation, shock resistance, and noise reduction [12,13,14]. Particle damping technology has been widely applied in the mechanical field. Park [15] studied the response of a mass spring damper under repeated impact. Skipor et al. [16] applied shock dampers to printing devices. Sims et al. [17] improved the stability of mechanical parts by utilizing the particle damper. Aiba et al. [18] introduced an impact damper with a variable impact force to eliminate vibration during metal cutting. Shah et al. [19] studied the effect of the particle nature of a particle damper with a piston on the damping property. Guo et al. [20] used the steady-state energy flow method to study the damping characteristics of a kind of particle damper with a piston. Zhang et al. [21] carried out a particle damper impact test, and the feasibility of dissipating impact kinetic energy via a particle damper was verified. Liu et al. [22] studied the design method of a particle damper for spoke structures, and the effective attenuation of umbrella-shaped vibration of spoke structures was realized.

Aimed at addressing the disadvantages of a low measurement accuracy and weak anti interference ability existing in general pressure sensors of the tension conversion pressure method, a kind of particle damping sensor is designed in this paper.

The other parts of this article are organized as follows: the working principle of the real-time tension monitoring system is introduced in Section 2. Section 3 describes the structure, the measuring principle, the simplified particle modelm and the principle of particle damping energy dissipation based on the discrete element method (DEM). The parameters of particles and spoke structure of the sensor are optimized by simulation software in Section 4. Section 5 includes the calibration test of the sensor, and a field experiment that is compared with the general pressure sensor. Finally, Section 6 concludes the research and discusses some future directions.

## 2. Real-Time Monitoring System for Wire Rope Tension of Multi-Rope Friction Hoist

**Figure 1:**
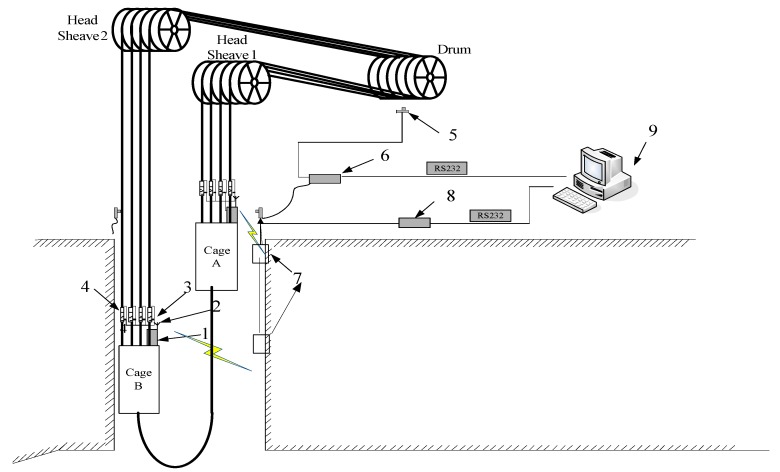
Schematic of tension monitoring system for multi-rope hoist. 1—Data acquisition module; 2—Wireless communication module; 3—Particle damping sensor; 4—Balance cylinder; 5—Hall sensor; 6—Position monitoring device; 7—Data receiver device; 8−RS485/232 converter; 9−Computer.

The real-time tension monitoring system of wire ropes is shown in Figure 1. Taking the cage B as an example, the working process is described as follows. The data acquisition module 1 is placed on the top of the cage to collect the signals acquired by particle damping sensor 3. Then, the modulated signals are transmitted through wireless communication module 2. The data receiver device 7 is installed in the pithead or shaft, which collects and transmits the signals from the data acquisition module 1. Finally, the signals are transmitted to the upper computer 9 and the tension monitoring is realized. Besides, the position monitoring device 6 is used to monitor the real-time rotation direction and rotation numbers of the drum by installing a Hall sensor 5 on the rotating shaft.

## 3. Sensor Design

### 3.1. Structure of Particle Damping Sensor

**Figure 2:**
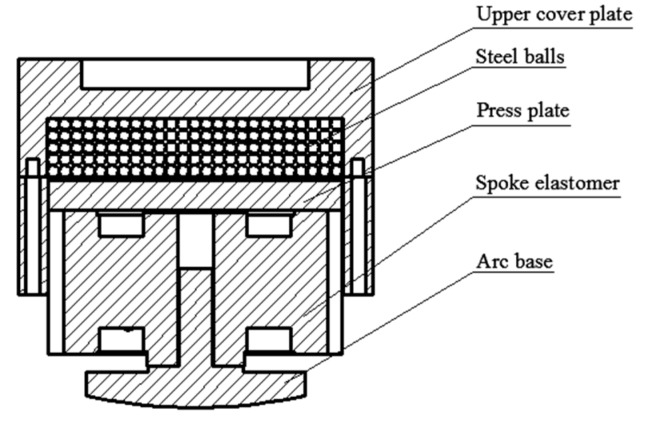
Schematic of particle damping sensor.

**Figure 3:**
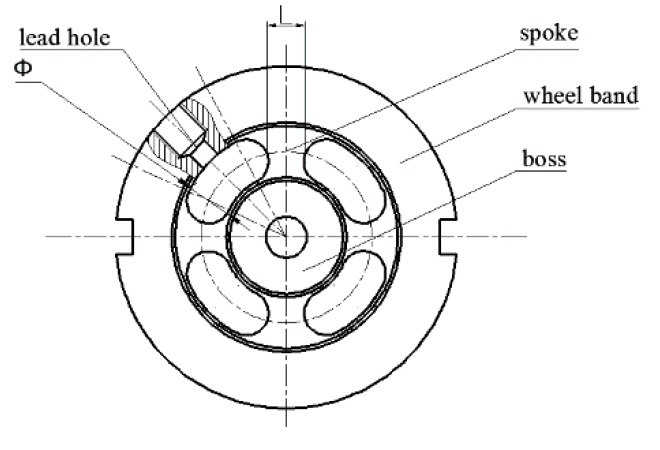
Spoke structure of sensor elastomer.

Figure 2 shows the structure of the particle damping sensor. The sensor is composed of an upper cover plate, steel balls, a cylindrical cavity, a press plate, a spoke elastomer, and an arc base. Lots of steel balls are placed in the cylindrical cavity. Considering the especial installation position of the sensor, its elastomer adopts the spoke structure, which can overcome the concentrated stress and distribute the pressure reasonably. Moreover, the structure has the characteristics of a strong anti-bias load, small size, and light weight [23,24]. As shown in Figure 3, the elastomer of the sensor is comprised of the wheel band, the boss, spokes, and the lead hole. When the sensor is compressed by an external force, the spoke is subjected to a shear stress and the load can be uniformly distributed. The stress can be measured by strain gauges attached on four spokes.

### 3.2. Measurement Principle of Particle Damping Sensor

**Figure 4:**
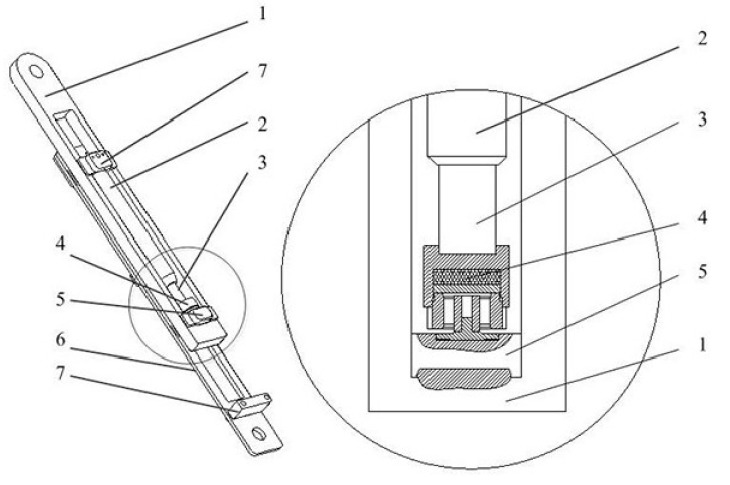
The hydraulic balancing cylinder model. 1—medium plate; 2—hydraulic cylinder; 3—piston rod; 4—particle damping sensor; 5—slide block; 6—side plate; 7—connecting pin.

The hydraulic balancing cylinder model is shown in Figure 4. The wire rope, the medium plate 1, and the slide block 5 are connected. The cage, the connecting pin 7, the hydraulic cylinder 2, and the side plate 6 are connected. The particle damping sensor 4 is placed between the piston rod 3 and slide block 5. Additionally, the sensor can move up and down with the slide block 5 in the slideway of side plate 6. The groove of the upper cover plate bears the pressure P from the piston rod 3, which is equal to the cage load. The pressure acts on the upper end face of the elastomer through the upper cover plate, the steel balls, and the pressure plate. Meanwhile, the pulling force of one wire rope, which is equal to its tension, acts on the arc base of the sensor 4 through the medium plate 1 and the slide block 5. Therefore, the arc base is subjected to a support force. The support force P’ acts on the lower end face of the elastomer through the arc base. When the acceleration of the sensor is neglected, the pressure P is approximately equal to the support force P’. That is, the wire rope tension can be obtained by measuring the pressure acting on the particle damping sensor.

A slight slip will be generated in the axial direction due to the nestification between the press plate and upper cover plate of the sensor when the wire ropes vibrate or are impacted. At this time, the collision and friction between the cylindrical cavity and the steel balls and among the steel balls both produce the particle damping effect. This not only effectively dissipates the vibration and load impact of wire ropes, but also solves the sudden jump of tension data. Therefore, acquired tension data become more stable and the filtering function of the sensor is realized.

### 3.3. Simplified Mechanical Model for Vibration Attenuation and Energy Dissipation of Particle Damping Sensor

In this section, forces among steel balls, and between steel balls and cylindrical cavity, are analyzed. It is assumed that the inner diameter of the cylindrical cavity where the steel balls are placed is D_1_. F represents the impacting force acting on the upper cover plate, N_1_ denotes the number of balls contacting with the upper cover plate, d_1_ represents the diameter of one steel ball, the pressure acting on one steel ball is q, and E means the elastic modulus of steel balls.

(1) Simplified Mechanical Model Among Steel Balls

**Figure 5:**
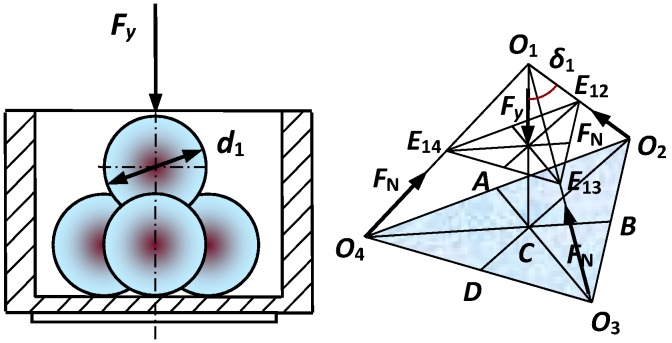
Simplified four-ball mechanical model.

The force among the steel balls in the cylindrical cavity can be simplified as a four-ball close contact model. As shown in Figure 5, *O*_1_, *O*_2_, *O*_3_, and *O*_4_ are the centers of four steel balls, respectively. F_N_ represents the supporting force between two balls and F_y_ represents the force along the vertical direction acting on ball 1. Assuming that *C* is the center of Δ*O*_2_*O*_3_*O*_4_, A, B, and D are the midpoints of three sides, respectively, and *δ*_1_ is the angle between *O*_1_*C* and *O*_1_*O*_2_.

The vertical force $F_{y}$ can be expressed as follows:

(1) $$F_{y}=F/N_{1}$$

According to the related theorem of triangle and mechanics, $\delta_{1}$ and $F_{N}$ can be deduced as

(2) $$\delta_{1}=\arctan\sqrt{2}/2$$

(3) $$F_{N}=F_{y}/(3\cos\delta_{1})=F_{y}/\sqrt{6}$$

The maximum contact force $q_{0}$ of steel balls can be expressed as

(4) $$q_{0}=0.388{\left[\frac{F_{y}E^{2}{\left(d_{1}/2+d_{1}/2\right)}^{2}}{{\left(d_{1}/2\right)}^{2}{\left(d_{1}/2\right)}^{2}}\right]}^{\frac{1}{3}}$$

The relative displacement ΔS of steel balls is described as

(5) $$\Delta S=1.23{\left[\frac{F_{y}^{2}(d_{1}/2+d_{1}/2)}{E^{2}(d_{1}/2)(d_{1}/2)}\right]}^{\frac{1}{3}}$$

The radius $r_{jc}$ of contact area of steel balls is expressed as

(6) $$r_{jc}=1.11{\left[\frac{F_{y}(d_{1}/2)(d_{1}/2)}{E(d_{1}/2+d_{1}/2)}\right]}^{\frac{1}{3}}$$

The work $W_{1}$ consumed by displacement ΔS can be written as

(7) $$W_{1}=2q_{0}(\pi r_{jc}^{2})\Delta S/3$$

(2) Simplified Mechanical Model Between the Steel Balls and Inner Cavity Wall 

**Figure 6:**
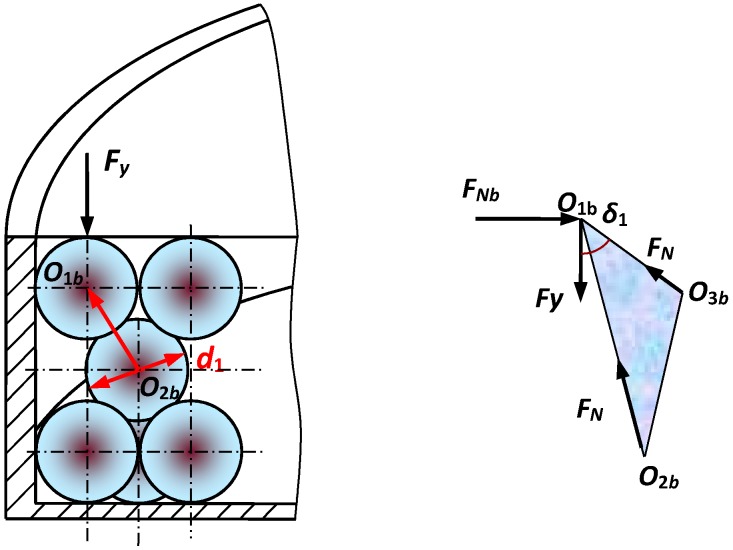
Simplified mechanical model between the steel balls and inner cavity wall.

The force between the steel balls and inner cavity wall can be simplified as the following model in Figure 6. The spherical centers of three balls are *O*_1*b*_, *O*_2*b*_, and *O*_3*b*_, respectively.

The extrusion force $F_{Nb}$ between the steel balls and inner cavity wall can be expressed as

(8) $$F_{Nb}=2F_{N}\sin\delta_{1}\cos(\pi /3)$$

The maximum contact stress between the steel balls and inner cavity wall can be described as

(9) $$q_{0b}=0.388{\left[\frac{F_{Nb}E^{2}{\left(-D_{1}/2+d_{1}/2\right)}^{2}}{{\left(D_{1}/2\right)}^{2}{\left(d_{1}/2\right)}^{2}}\right]}^{\frac{1}{3}}$$

The relative displacement $\Delta S_{b}$ between the steel balls and inner cavity wall can be acquired as

(10) $$\Delta S_{b}=1.23{\left[\frac{F_{Nb}^{2}(-D_{1}/2+d_{1}/2)}{E^{2}(-D_{1}/2)(d_{1}/2)}\right]}^{\frac{1}{3}}$$

The radius of the contact area $r_{jcb}$ between the steel balls and inner cavity wall is

(11) $$r_{jcb}=1.11{\left[\frac{F_{Nb}(-D_{1}/2)(d_{1}/2)}{E(-D_{1}/2+d_{1}/2)}\right]}^{\frac{1}{3}}$$

The work $W_{2}$ consumed by displacement $\Delta S_{b}$ can be deduced as

(12) $$W_{2}=2q_{0b}(\pi r_{jcb}^{2})\Delta S_{b}/3$$

### 3.4. Particle Damping Model and the Energy Dissipation Principle Based on DEM

The goal of DEM is to separate a discontinuous body into a set of rigid elements. Then, the motion equations of each rigid element are established on the basis of the relationship between force and displacement. Finally, these motion equations are solved iteratively by the dynamic relaxation method, and thus the whole motion form of the discontinuous body is obtained. So far, common particle contact models based on DEM include the viscous-elastic contact model and viscous-elastic-plastic contact model. The study on the viscous-elastic contact model is more mature. Therefore, we adopt the viscous-elastic contact model in this paper.

(1) Mechanical Contact Model Between Steel Balls Based on DEM

**Figure 7:**
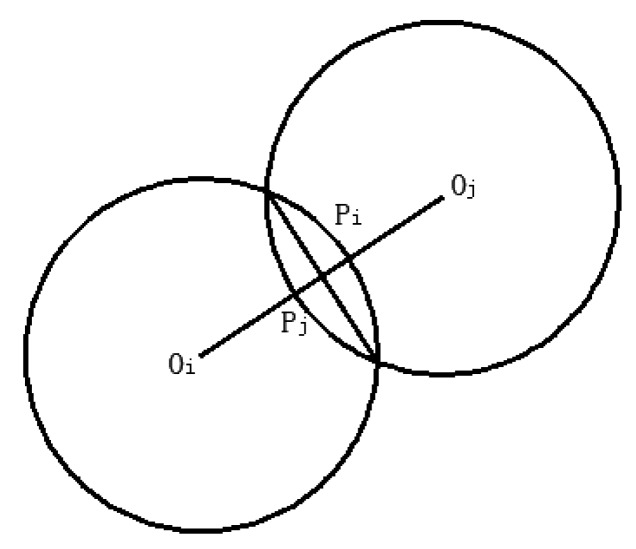
The interaction model between the steel ball i and the steel ball j.

The interaction model between the steel ball *i* and the steel ball *j* is shown in Figure 7. The sphere centers of two balls are *O_i_* and *O_j_*, respectively. The line segment *O_i_O_j_* is intersected with two balls at $P_{i}$ and $P_{j}$, respectively. Two balls will contact each other when the length of *O_i_O_j_* is less than the sum of the radius of two balls. That is:

(13) $$D_{ij}<R_{i}+R_{j}$$

where $D_{ij}$ represents the length of line segment *O_i_O_j_*; and $R_{i}$ and $R_{j}$ are the radius of the steel ball *i* and *j*, respectively. 

The relative motion between two balls is mainly decomposed into tangential motion and normal motion. Additionally, the normal force and the tangential force cause energy dissipation. 

The normal superposition can be expressed as

(14) $$\delta_{n}=R_{i}+R_{j}-D_{ij}$$

The normal component $f_{n}$ and tangential component $f_{t}$ of contact force can be described as

(15) $$f_{n}=f_{n1}+f_{n2}=k_{n}\delta_{n}^{2/3}+c_{n}\delta_{n}^{1/4}\overset{\cdot }{\delta_{n}}$$

(16) $$f_{t}=-\mu f_{n}\overset{\cdot }{\delta_{t}}/\left|\overset{\cdot }{\delta_{t}}\right|$$

where $f_{n1}$ is the normal elastic force; $f_{n2}$ is the normal viscous force; $\overset{\cdot }{\delta_{n}}$ represents the velocity of ball i relative to ball j; $\overset{\cdot }{\delta_{t}}$ represents the tangential velocity; $c_{n}$ and μ are the damping constant and friction coefficient among steel balls and between the balls and the inner cavity wall, respectively; and $k_{n}$ is the elastic coefficient.

(17) $$k_{n}=\frac{\sqrt{2R}}{3}\frac{E_{p}}{1-v_{p}^{2}}$$

where E and v mean the elastic modulus and Poisson’s ratio, respectively. The subscript *p* represents the particle.

(2) Mechanical Contact Model Between the Ball and the Cavity Wall Based on DEM

**Figure 8:**
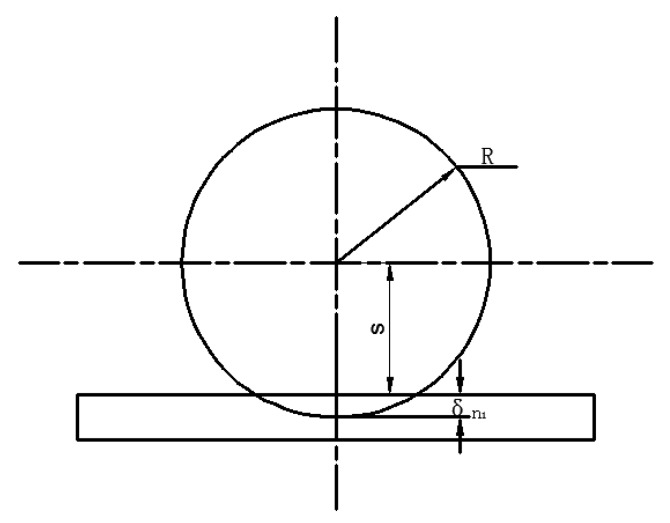
The interaction model between steel balls and the cavity wall.

Figure 8 shows the contact model between the ball and the cavity wall. s means the distance between the ball center and the cavity wall, and R is the radius of each ball. Similarly, the normal force and tangential force can be expressed as Equations (15) and (16), respectively. Under this circumstance, the elastic coefficient can be expressed as

(18) $$k_{n1}=\frac{4\sqrt{R}}{3}\frac{E_{p}E_{0}}{\left(1-v_{p}^{2}\right)E_{0}+\left(1-v_{0}^{2}\right)E_{p}}$$

where the subscript 0 represents the inner cavity wall. In this case, the normal superposition $\delta_{n1}$ is

(19) $$\delta_{n1}=R-s$$

(3) Energy Dissipation Principle

Inelastic contact collision energy dissipation and friction energy dissipation are two main ways to dissipate energy for particle damping. The energy dissipation produced by particle contact mainly includes normal energy dissipation and tangential energy dissipation. For the normal energy dissipation, the normal elastic contact force $f_{n1}$ does not consume energy, and only the normal viscous force $f_{n2}$ consumes energy. The energy dissipation by normal force in time Δt can be calculated as

(20) $$E_{nLos}=\sum_{m=1}^{IC}\left(f_{n2m}\cdot \Delta r_{m,n}\right)=\sum_{m=1}^{IC}[-C_{nm}(v_{i,n}-v_{jm,n})\cdot (v_{i,n}-v_{jm,n})\Delta t]$$

where IC means the number of contact pairs at some point; $\Delta r_{m,n}$ is the normal relative displacement vector of the *m*th contact pair in this period; $C_{n}$ means the normal viscous damping coefficient; $v_{i}$ and $v_{j}$ represent the speed of balls *i* and *j*, respectively; and $v_{i,n}$ and $v_{j,n}$ are the projection on the normal direction of $v_{i}$ and $v_{j}$, respectively.

The tangential friction exists at the tangential direction of particle contact. The energy dissipation in the tangential direction can be divided into two cases according to the Mohr-Coulomb friction theorem.

(21) $$if\left|F_{t}^{n+1}\right|<\mu_{t}\left|F_{n}^{n+1}\right|$$

(22) $$E_{tvLos}=\sum_{m=1}^{IC}[-c_{t}(v_{ik,t}-v_{jk,t})\cdot (v_{ik,t}-v_{jk,t})\Delta t]$$

(23) $$if\left|F_{t}^{n+1}\right|\geq \mu_{t}\left|F_{n}^{n+1}\right|$$

(24) $$E_{tsLos}=\sum_{m=1}^{IC}(-\mu_{t}\left|F_{n}^{n+1}\right|\left|v_{ik,t}-v_{jk,t}\right|\Delta t)$$

where $F_{n}^{n+1}$ and $F_{t}^{n+1}$ mean the normal force and the estimation value of tangential force, respectively, at t time; $E_{tvLos}$ and $E_{tsLos}$ represent the tangential viscous force energy dissipation and friction force at the tangential direction energy dissipation, respectively; $v_{ik,t}$ and $v_{jk,t}$ are the projection in the tangential direction of $v_{i}$ and $v_{j}$, respectively; $c_{t}$ is the tangential viscous damping coefficient; and $\mu_{t}$ is the static sliding friction coefficient.

The energy dissipation at the tangential direction in time Δt can be calculated as

(25) $$E_{tLos}=E_{tvLos}+E_{tsLos}$$

The total energy dissipation of the system can be expressed as

(26) $$E_{loss}=E_{nLos}+E_{tLos}$$

## 4. Simulation Analysis of the Sensor

In this section, the structure of the spoke elastomer is modeled and its design parameters are optimized. Moreover, modal analysis of the structure is carried out by Ansys. Finally, the optimum structural parameters can be obtained by the orthogonal test.

(1) Structural Optimization of the Spoke Elastomer

**Figure 9:**
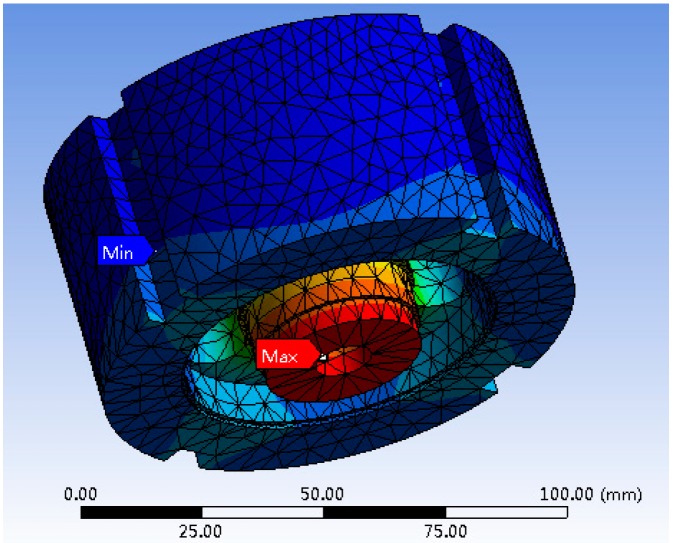
Displacement cloud image.

**Figure 10:**
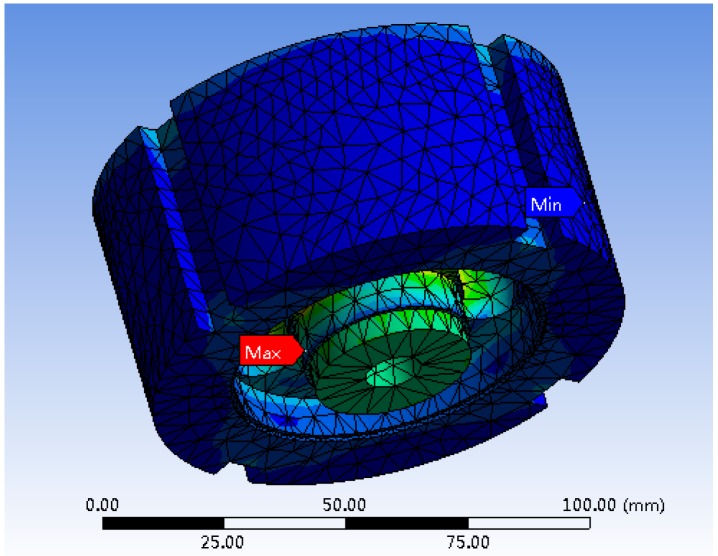
Stress cloud image.

The displacement and stress cloud images of the elastic body are achieved through software simulation, shown in Figure 9 and Figure 10, respectively.

As shown in Figure 9, it can be seen that the maximum displacement occurs in the middle position of the wheel boss bottom, and the size is 1.8612e−002 mm. The minimum displacement occurs in the underside of the wheel hoop, and the size is 0mm. Figure 10 shows that the maximum stress occurs at the junction between the wheel boss and the spoke bottom, which is equal to 150.53 MPa. The minimum stress occurs on the side surface of the wheel boss, and the value is 9.1505e−002 MPa.

(2) Analysis of Orthogonal Test

The sensor is used to measure the static axial force. In this test, axial sensitivity S and natural frequency K of the sensor are selected as test parameters. The product W of S and K is the test index. The greater the product W, the better the comprehensive performance. Three structural parameters of the elastomer structure, L, Φ, and H, are selected to evaluate the performance of the sensor. L and Φ are shown in Figure 3, where H represents the height of the spoke. Taking the installation conditions of the sensor into account, it is necessary to select an appropriate sensor size. In this test, we adopt the L_9_3^4^ orthogonal table and the level of each factor is set to 3. The results of the orthogonal table are shown in Table 1.

**Figure 11:**
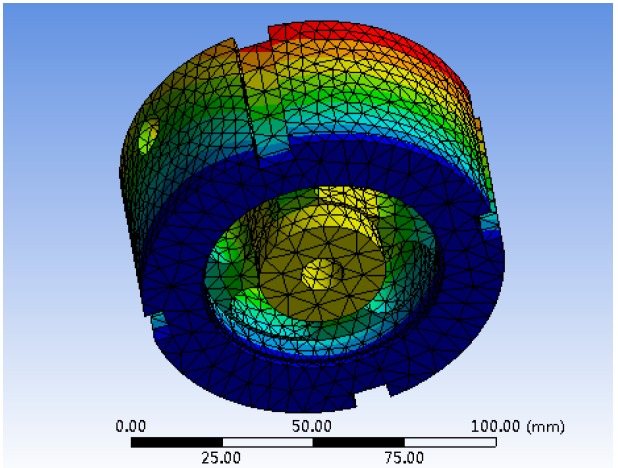
The resonance frequency of the first-order modal.

**Figure 12:**
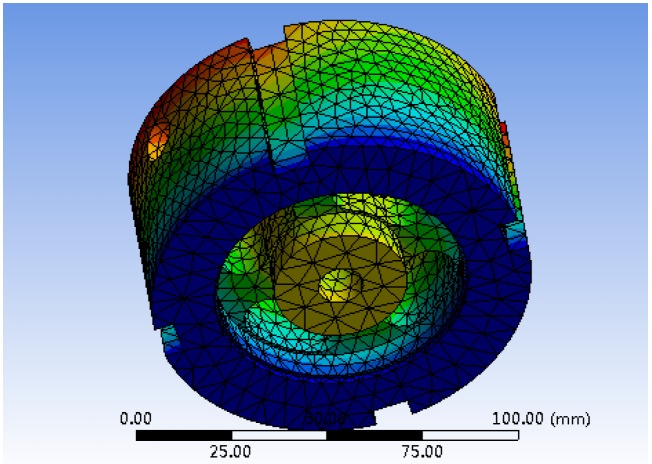
The resonance frequency of the second-order modal.

The axial sensitivity in the orthogonal table is obtained by static analysis and calculation of the model, which represents the ratio of the strain produced by the elastomer under the action of external force to the load. The natural frequency of the elastic body can be obtained after model importing and modal analysis. The first and second order natural frequencies modal graphs are shown in Figure 11 and Figure 12, respectively.

As shown in Table 1, the value W of test 8 is the largest, and corresponding L, Φ, and H values are 14 mm, 11.2 mm, and 29 mm, respectively. At this time, the sensor will achieve the optimum performance.

### 4.1. Simulation of Vibration Reduction and Filtering Effect Based on Particle Damping Parameters

**Figure 13:**
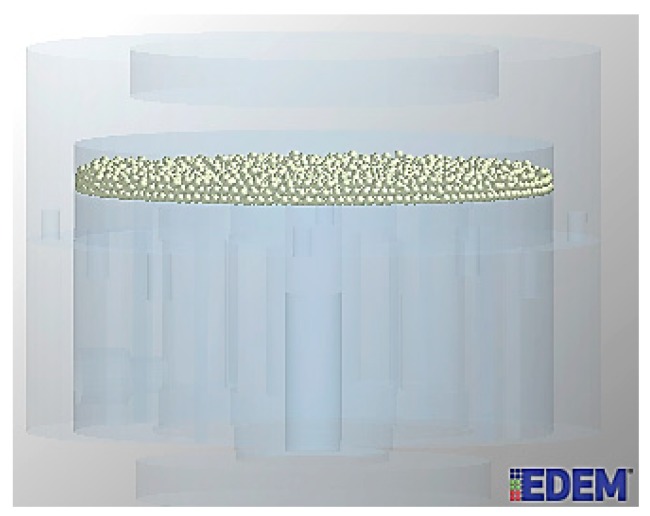
The model of particle damping sensor in EDEM.

According to the introduction and analysis in Section 3, steel balls in the cylindrical cavity of the sensor will produce a damping effect when the wire ropes fluctuate violently. In this section, EDEM software is used to simulate the change regulation of normal force, tangential force, and kinetic energy with time under a large filling rate and different ball diameters. Then, the optimum ball parameters are determined. The larger the normal force between two particles is, the greater the collision degree and energy dissipation is. Similarly, the larger the tangential force is, the greater the friction energy dissipation is, and the better the vibration reduction effect is. Moreover, the larger the kinetic energy between particles is, the more obvious the relative movement is. Therefore, the collision opportunities become more and the particle damping effect is better. The model of the particle damping sensor in EDEM is shown in Figure 13.

#### 4.1.1. Effect of Particle Diameter on Damping Effect

According to the actual situation in the field and the theoretical analysis in Section 3.3, the normal force, tangential force, and kinetic energy of steel balls with diameters of 2 mm and 3 mm are simulated under the filling rate of 95%.

(1) Normal Force

**Figure 14:**
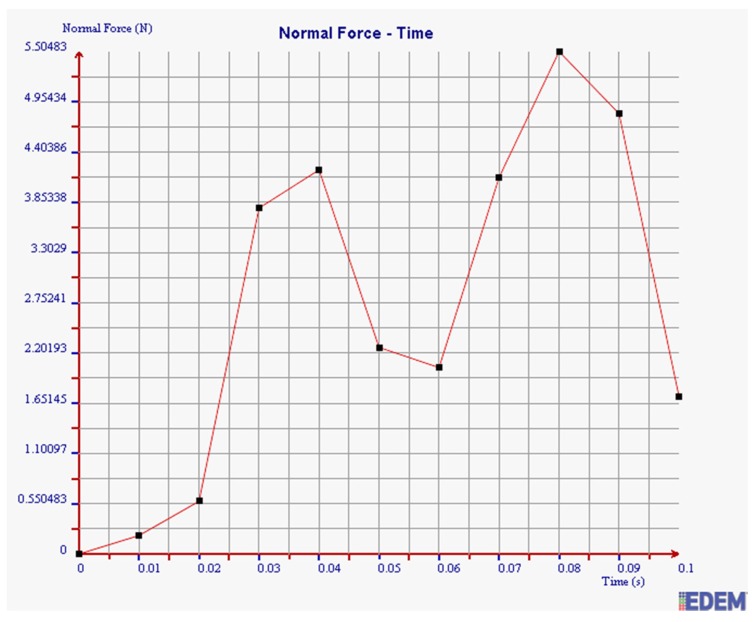
Normal force of 2 mm steel balls.

**Figure 15:**
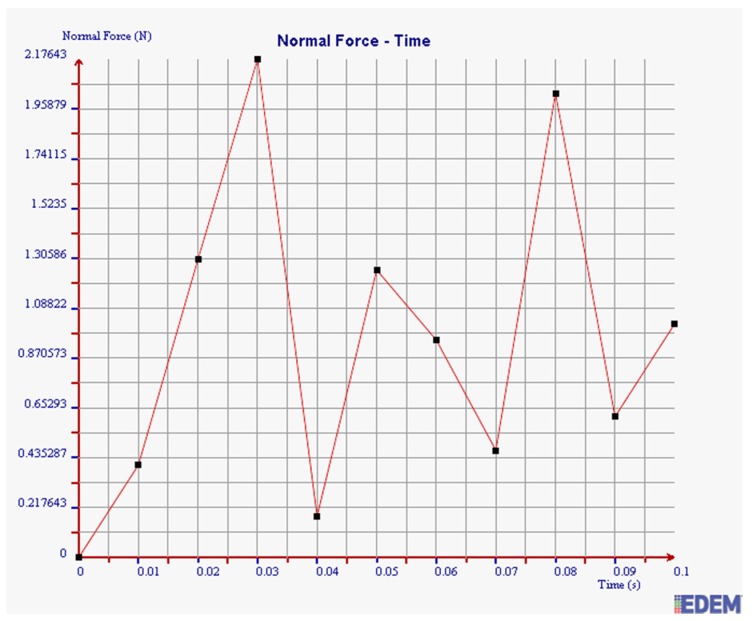
Normal force of 3 mm steel balls.

As shown in Figure 14 and Figure 15, the maximum normal force of 2 mm steel balls within 0.1 s is 5.51 N, and the value of 3 mm steel balls is 2.18 N. The maximum normal force of 2 mm steel balls is 2.53 times more than that of 3 mm balls. 

(2) Tangential Force

**Figure 16:**
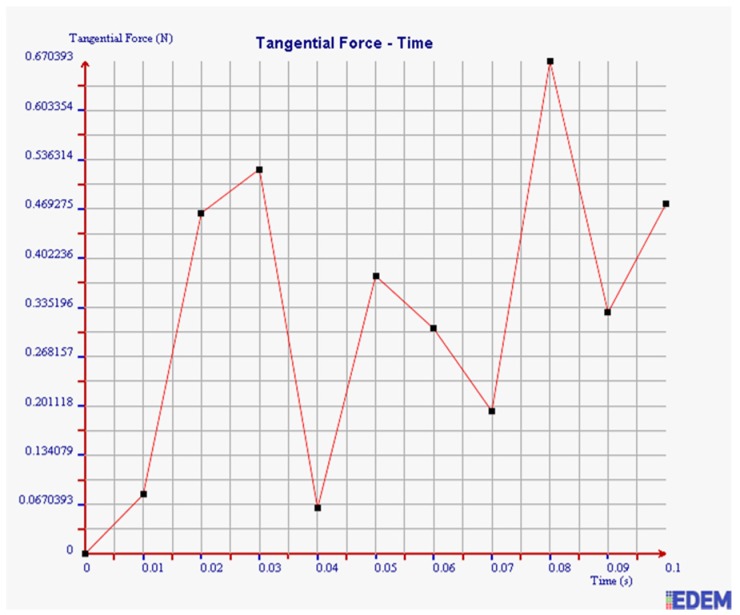
Tangential force of 2 mm steel balls.

**Figure 17:**
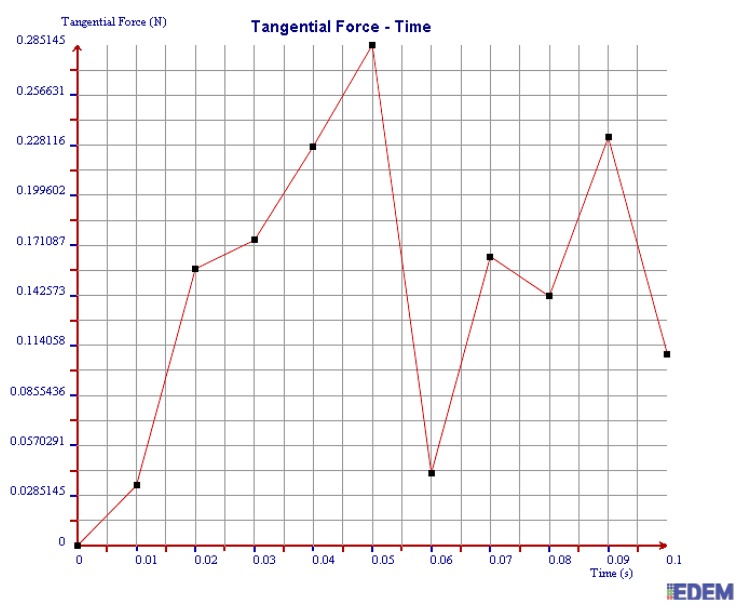
Tangential force of 3 mm steel balls.

It can be seen from Figure 16 and Figure 17 that the maximum tangential force of 2 mm steel balls within 0.1 s is 0.67 N, and the value of 3 mm steel balls is 0.28 N. The maximum tangential force of 2 mm steel balls is 2.39 times more than that of 3 mm balls.

(3) Kinetic Energy

**Figure 18:**
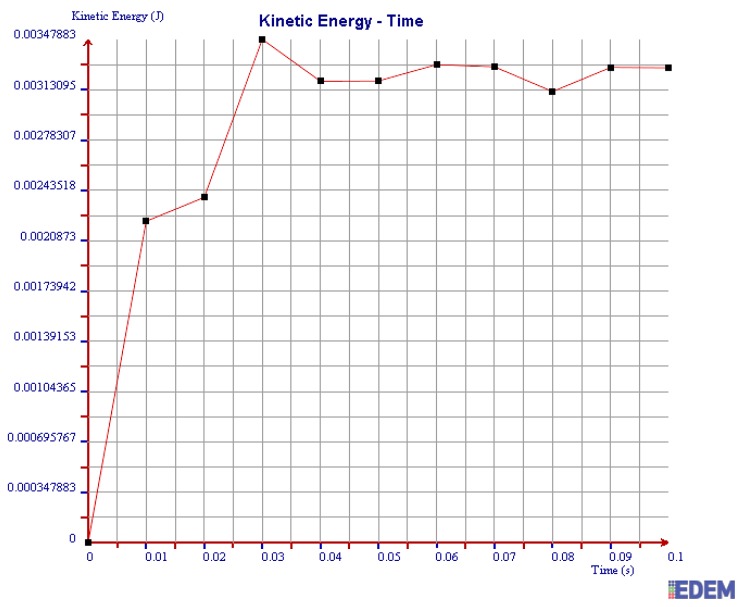
Kinetic energy of 2 mm steel balls.

**Figure 19:**
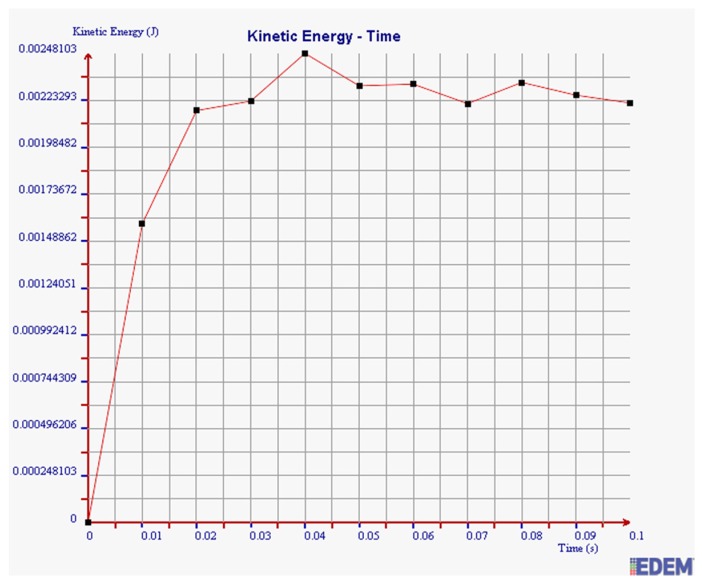
Kinetic energy of 3 mm steel balls.

It can be seen from Figure 18 and Figure 19 that the maximum kinetic energy of 2 mm steel balls within 0.1 s is 0.00348 J, and the value of 3 mm steel balls is 0.00248 J. The maximum tangential force of 2 mm steel balls is 1.4 times more than that of 3 mm balls.

In summary, the maximum normal force, tangential force, and kinetic energy of 2 mm steel balls are all greater than those of 3 mm steel balls within 0.1 s. It is illustrated that the collision degree and the friction energy dissipation of 2 mm steel balls are greater under the same test conditions. The sensor can achieve better vibration attenuation and energy dissipation by using the 2 mm steel balls.

#### 4.1.2. Influence of Particle Materials on Damping Effect

In this section, the normal force, tangential force, and kinetic energy of chromium balls with a diameter of 2 mm are simulated, respectively.

**Figure 20:**
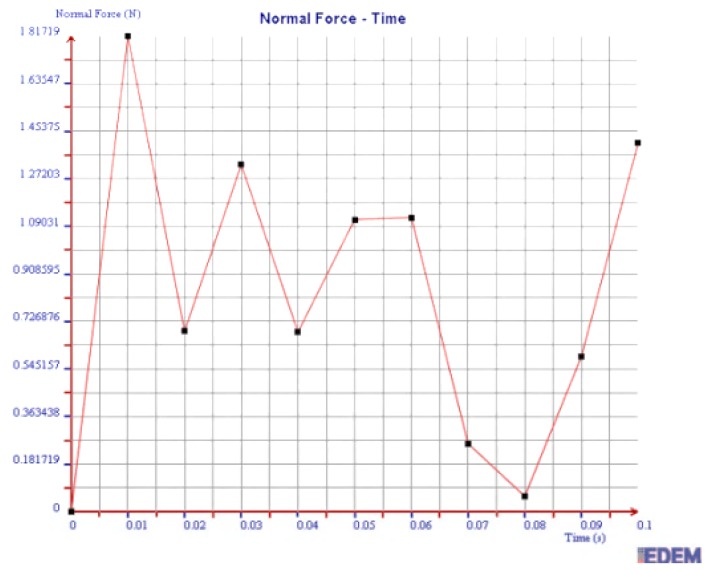
Normal force of 2 mm chromium balls.

**Figure 21:**
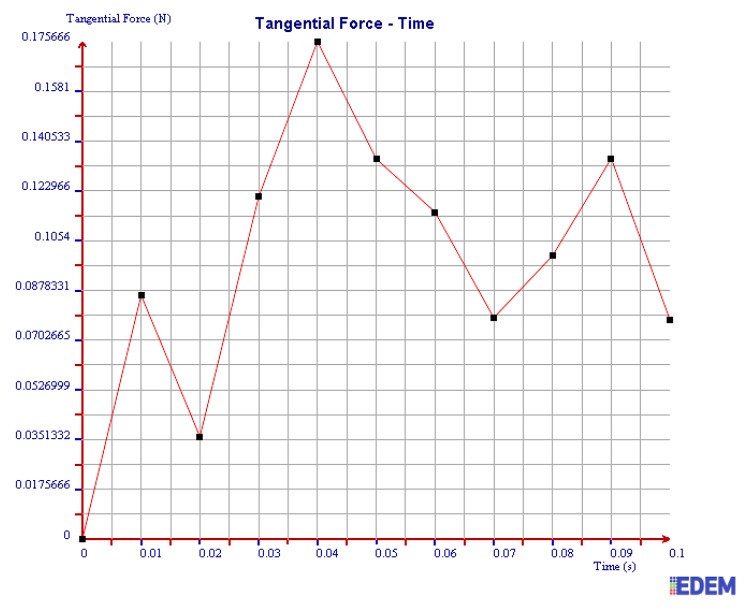
Tangential force of 2 mm chromium balls.

**Figure 22:**
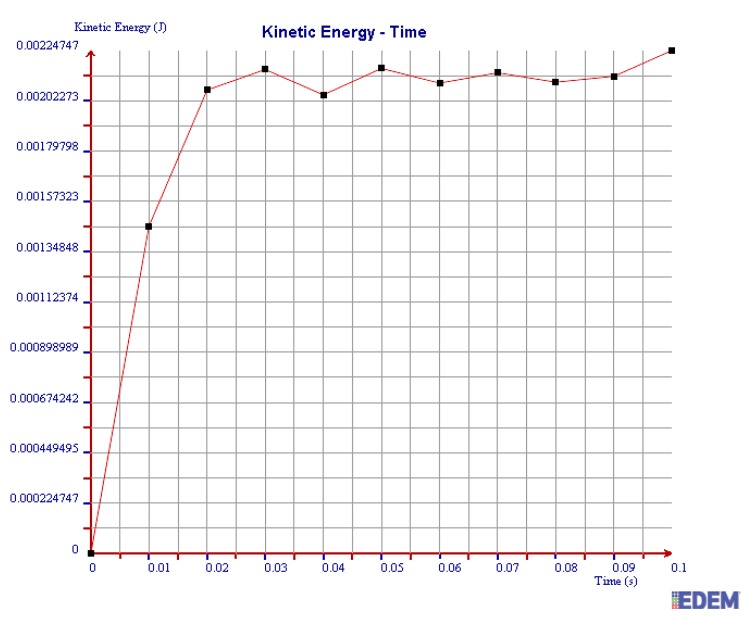
Kinetic energy of 2 mm chromium balls.

As can be seen in Figure 20, Figure 21 and Figure 22, the maximum values of normal force, tangential force, and kinetic energy of 2 mm chromium balls within 0.1 s are 1.82 N, 0.17 N, and 0.00225 J respectively, which are smaller than those of 3mm steel balls. Additionally, the maximum values of normal force, tangential force, and kinetic energy of 2 mm steel balls within 0.1 s are 3 times, 3.94 times, and 1.55 times more than those of 2 mm chromium balls, respectively.

Through comparing the maximum normal force, tangential force, and kinetic energy within 0.1 s of 2 mm steel balls, 3 mm steel balls, and 2 mm chromium balls, the values of 2 mm steel balls are the largest. Therefore, the steel balls of 2 mm can dissipate more energy and have a better vibration reduction effect.

## 5. Calibration and Field Test of the Sensor

**Figure 23:**
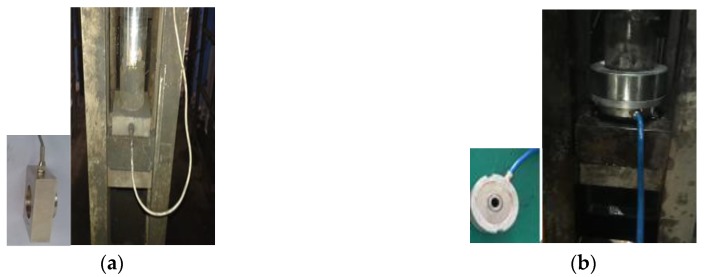
Installation situation. (**a**) General pressure sensor; (**b**) Particle damping sensor.

The sensor prototype was designed and manufactured based on the optimized parameters in Section 4. Then, the calibration experiment was carried out in the laboratory, and the comparison tests of the sensor were completed in a coal mine. Compared with the general pressure sensor, high-frequency components of tension signals measured by the particle damping sensor are effectively reduced, and accurate monitoring of wire rope tension is realized. Field installation situations of the general pressure sensor and the particle damping sensor are shown in Figure 23.

### 5.1. Static Calibration of the Sensor

**Figure 24:**
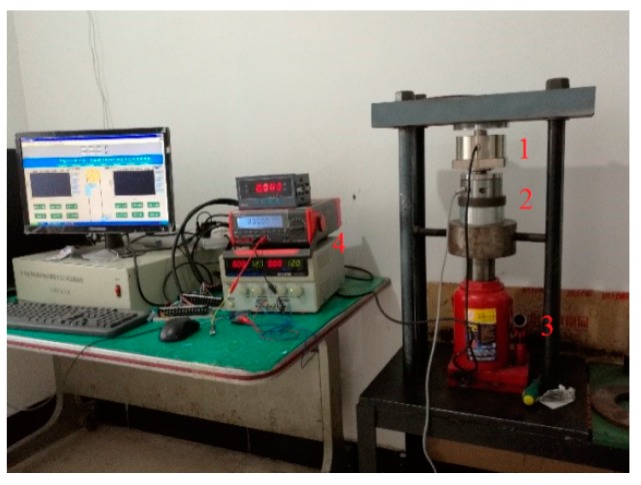
Experimental setup. (**1**) Particle damping sensor; (**2**) Standard sensor; (**3**) Hydraulic cylinder; (**4**) Displayers.

**Figure 25:**
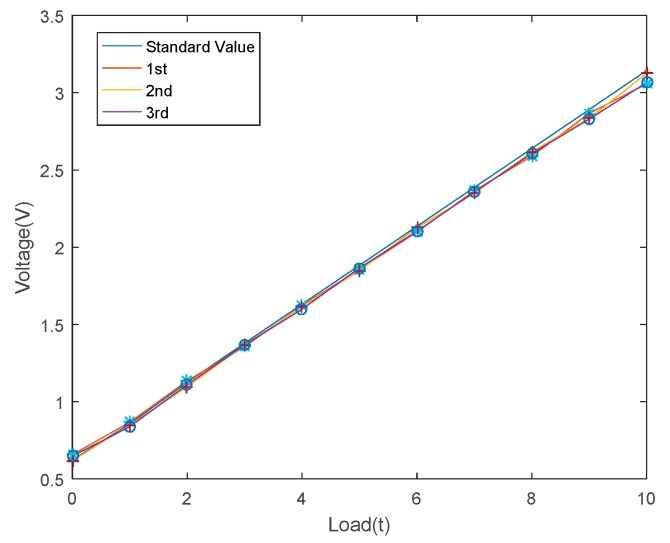
Linearity experiment results of the sensor.

In order to check the linearity of the sensor, measured data of the standard sensor and designed sensor are compared. The experimental devices are shown in Figure 24. The hydraulic cylinder and the two sensors are stacked concentrically. Additionally, the outputs of the particle damper sensor are voltage values. Theoretically, the output voltage of the sensor is proportional to the external force. In the experiment, the pressure value of the hydraulic cylinder was loaded from 0 ton to 10 tons and the experiment was repeated three times. Corresponding experimental results are shown in Figure 25. It can be seen from the figure that the designed sensor has high linearity and can be applied in coal mines.

### 5.2. Field Application of the Sensor

(1) Measurement and Analysis of Wire Rope Tension

**Figure 26:**
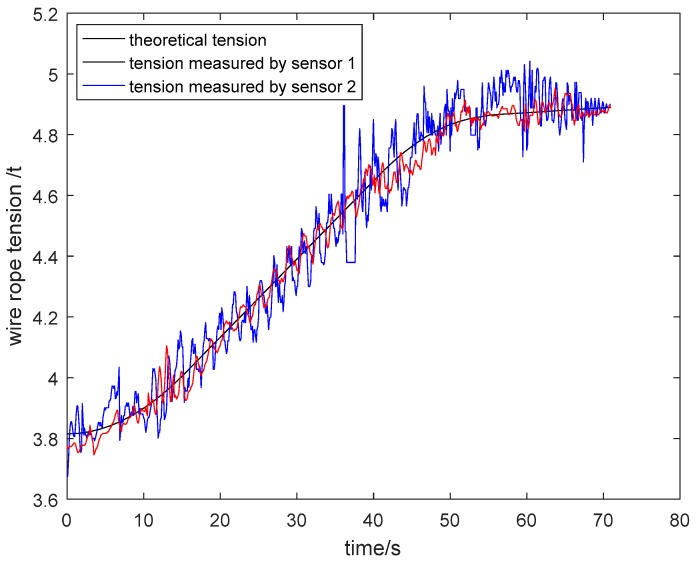
Tension curves during lifting process of cage without load.

**Figure 27:**
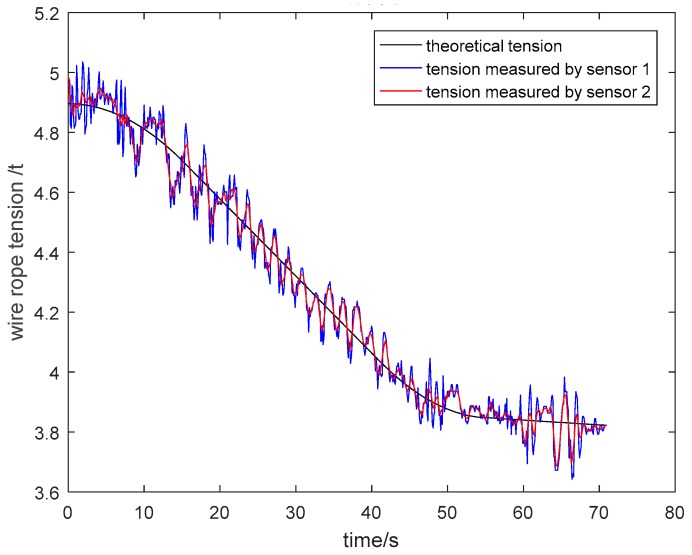
Tension curves during descending process of cage without load.

**Figure 28:**
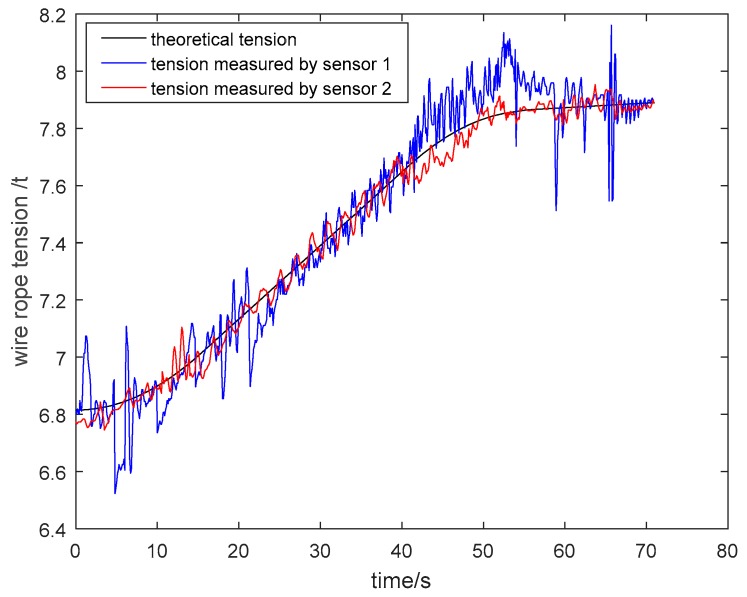
Tension curves during lifting process of cage with load.

**Figure 29:**
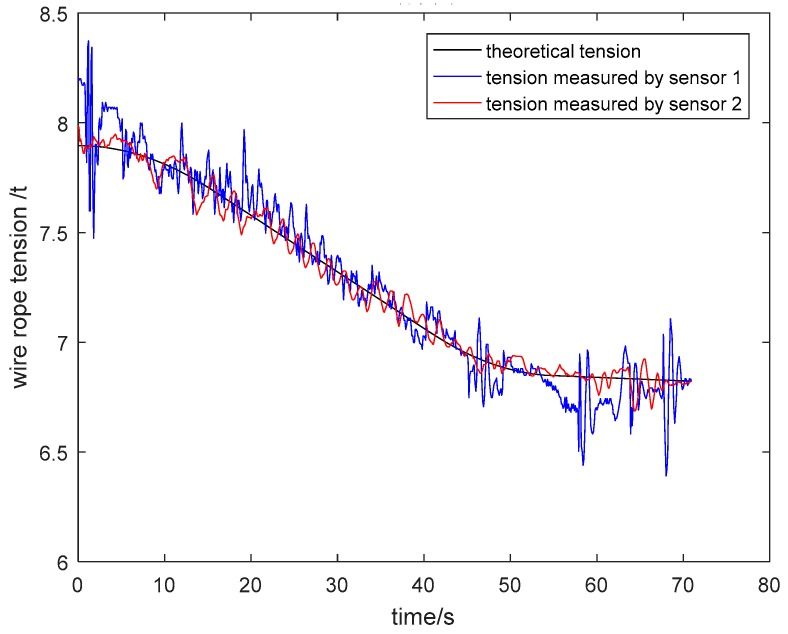
Tension curves during descending process of cage with load.

The particle damping sensor was installed in a four-rope friction hoist. Acquired tension signals of wire ropes were analyzed and processed in the upper computer. As shown in Figure 26 and Figure 27, the tension curves measured by general pressure sensor 1 and particle damping sensor 2, and the theoretical tension curve varying with the time of cage lifting and descending without load, are drawn in the same coordinate system. Similarly, the tension curves of the cage with load are shown in Figure 28 and Figure 29.

As shown in Figure 26, Figure 27, Figure 28 and Figure 29, the tension curves in the lifting process all take on an ascending trend. Similarly, the tension curves in the descending process all take on a downward trend. Besides, the fluctuation amplitude of tension signals is different at different lifting or descending stages. The signals are relatively stable in the process of uniform running, and the fluctuation is larger in the process of accelerating or decelerating due to relatively complex working conditions at these stages.

Taking the lifting process of the cage without load as an example, the vibration of wire ropes is intense from 0 s to 15 s because the system needs to overcome the lifting load and inertia load at this stage. During the uniform speed period, the tension fluctuation of wire ropes is stable because energy stored at the acceleration stage is released. At the deceleration stage from 36 s to 54 s, the tension varies greatly. On the one hand, this is because that the energy stored at the acceleration and uniform speed stage is released more fully. On the other hand, the unevenness of the cage guide may aggravate the vibration of wire ropes.

The density of tail rope used in the field is 6.64 kg/m and the length of a tail rope from shaft bottom to pithead is about 330 m. Thus, the changed mass of two tail ropes is about 4.38 tons. As can be seen from the tension curve measured by the particle damping sensor 2 in Figure 26, the tension variation of one wire rope from shaft bottom to pithead is about 1.1 tons. As a result, the total tension variation of four wire ropes is about 4.4 tons, which is approximately equal to the changed mass of two tail ropes.

The above analysis shows that the designed particle damping sensor can achieve tension monitoring of wire ropes.

(2) Noise Analysis of the Measurement Value of Two Kinds of Sensors

**Figure 30:**
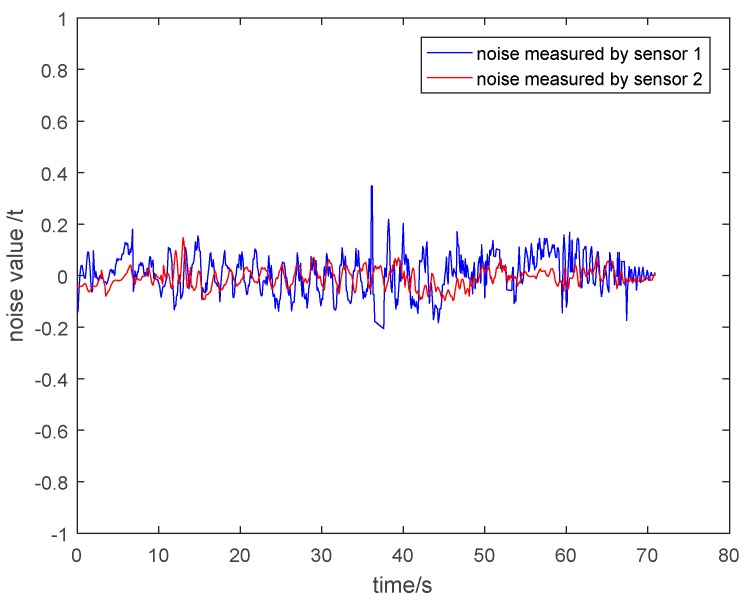
Noise value curves during lifting process of cage without load.

**Figure 31:**
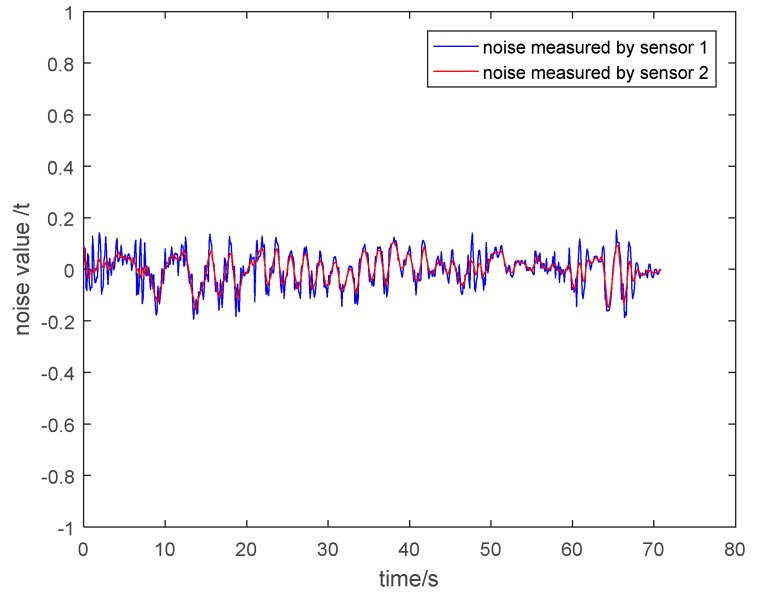
Noise value curves during descending process of cage without load.

**Figure 32:**
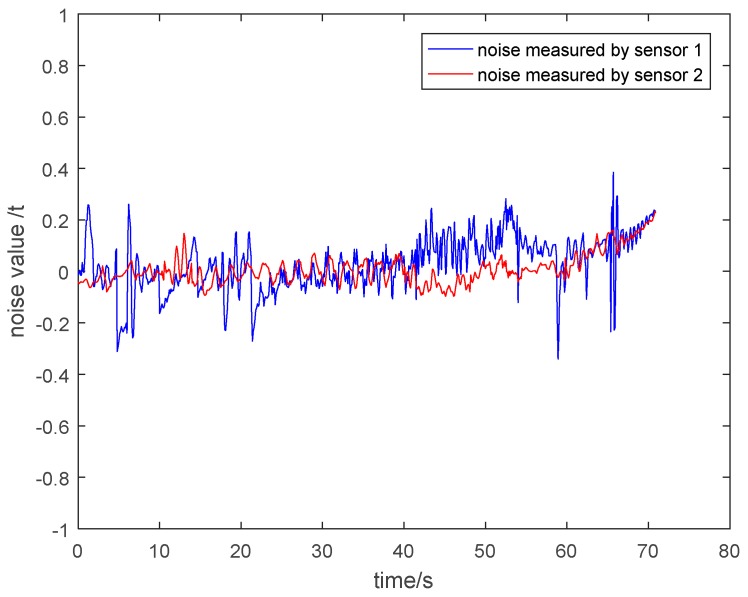
Noise value curves during lifting process of cage with load.

**Figure 33:**
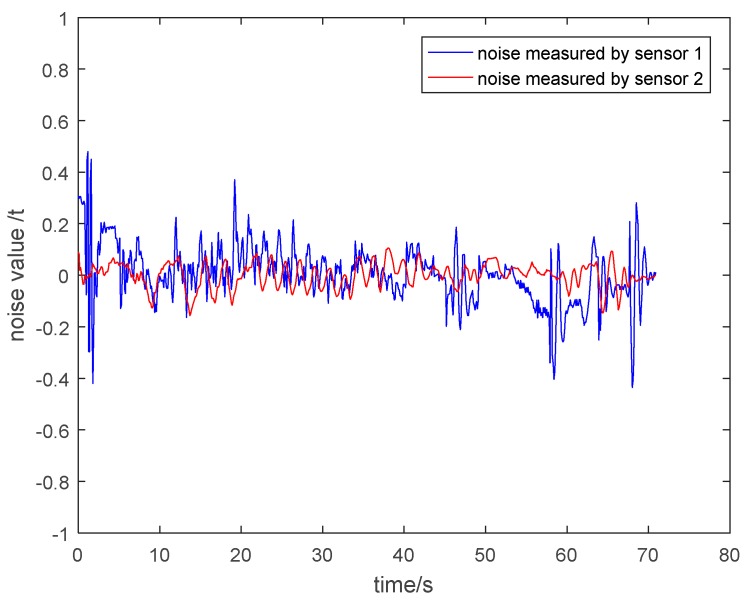
Noise value curves during descending process of cage with load.

In order to clearly explain that the particle damping sensor 2 has a better vibration and noise reduction effect than the general pressure sensor 1, it is necessary to show the noise of the measurement value of two kinds of sensors. The noise is equal to the value of subtracting the theoretical tension from the measured tension. The noise under different circumstances is shown in Figure 30, Figure 31, Figure 32 and Figure 33. It is shown that the noise of the measurement value of the particle damping sensor 2 is smaller when comparing it with the general pressure sensor 1 in the whole running process of the cage with or without load.

Through the comparison and analysis of Figure 26, Figure 27, Figure 28, Figure 29, Figure 30, Figure 31, Figure 32 and Figure 33, it is concluded that the tension signals measured by general pressure sensor 1 contain more high-frequency components, which cannot effectively characterize the actual lifting load. However, the tension fluctuation amplitude measured by the particle damping sensor 2 is relatively small, and the measured signals are more stable and closer to the theoretical tension.

## 6. Conclusions and Future Work

This paper presents a kind of particle damping sensor with the function of vibration dissipation. The force condition of steel balls in practice is simplified and analyzed, and the energy dissipation principle based on DEM is introduced. Then, the relevant parameters of the spoke structure and filled particles of the sensor are simulated and optimized. Finally, the designed sensor based on optimized parameters is tested in a coal mine, and the tension data measured by the general pressure sensor and particle damping sensor, and the theoretical tension values are compared. The results show that the designed sensor can effectively reduce high-frequency components of tension signals and achieve more accurate tension measurement.

In future research, the viscous-elastic-plastic contact model and the influence of plastic deformation on energy consumption will be further studied. Moreover, in order to achieve a more concise and accurate expression of the particle damping effect, the damping force will be discussed as an evaluation parameter.

## Figures and Tables

**Table 1:** Results of the orthogonal table.

	Factors	Φ	L	H	S	K	W = S × K
Test Numbers							
1		12	10.2	27	0.0144	10639	152.769
2		12	11.2	29	0.0158	10789	170.858
3		12	12.2	31	0.0126	10930	137.631
4		13	10.2	27	0.0161	10619	169.904
5		13	11.2	29	0.0159	10769	171.447
6		13	12.2	31	0.0149	10914	163.548
7		14	10.2	27	0.0169	10604	178.951
8		14	11.2	29	0.0174	10755	186.778
9		14	12.2	31	0.0162	10902	177.024
